# Associations of Retinal Microvascular Diameters and Tortuosity With Blood Pressure and Arterial Stiffness: United Kingdom Biobank

**DOI:** 10.1161/HYPERTENSIONAHA.119.13752

**Published:** 2019-10-28

**Authors:** Robyn J. Tapp, Christopher G. Owen, Sarah A. Barman, Roshan A. Welikala, Paul J. Foster, Peter H. Whincup, David P. Strachan, Alicja R. Rudnicka

**Affiliations:** 1 From the Population Health Research Institute, St George’s University of London, United Kingdom (R.J.T., C.G.O., P.H.W., D.P.S., A.R.R.); 2 Melbourne School of Population and Global Health, University of Melbourne, Australia (R.J.T.); 3 Faculty of Science, Engineering and Computing, Kingston University, Surrey, United Kingdom (S.A.B., R.A.W.); 4 Integrative Epidemiology Research Group, UCL Institute of Ophthalmology, United Kingdom (P.J.F.); 5 NIHR Biomedical Research Centre at Moorfields Eye Hospital, United Kingdom (P.J.F.).

**Keywords:** blood pressure, cardiovascular diseases, hypertension, microvasculature, tortuosity

## Abstract

To examine the baseline associations of retinal vessel morphometry with blood pressure (BP) and arterial stiffness in United Kingdom Biobank. The United Kingdom Biobank included 68 550 participants aged 40 to 69 years who underwent nonmydriatic retinal imaging, BP, and arterial stiffness index assessment. A fully automated image analysis program (QUARTZ [Quantitative Analysis of Retinal Vessel Topology and Size]) provided measures of retinal vessel diameter and tortuosity. The associations between retinal vessel morphology and cardiovascular disease risk factors/outcomes were examined using multilevel linear regression to provide absolute differences in vessel diameter and percentage differences in tortuosity (allowing within person clustering), adjusted for age, sex, ethnicity, clinic, body mass index, smoking, and deprivation index. Greater arteriolar tortuosity was associated with higher systolic BP (relative increase, 1.2%; 95% CI, 0.9; 1.4% per 10 mmHg), higher mean arterial pressure, 1.3%; 0.9, 1.7% per 10 mmHg, and higher pulse pressure (PP, 1.8%; 1.4; 2.2% per 10 mmHg). Narrower arterioles were associated with higher systolic BP (−0.9 µm; −0.94, −0.87 µm per 10 mmHg), mean arterial pressure (−1.5 µm; −1.5, −1.5 µm per 10 mmHg), PP (−0.7 µm; −0.8, −0.7 µm per 10 mmHg), and arterial stiffness index (−0.12 µm; −0.14, −0.09 µm per ms/m^2^). Associations were in the same direction but marginally weaker for venular tortuosity and diameter. This study assessing the retinal microvasculature at scale has shown clear associations between retinal vessel morphometry, BP, and arterial stiffness index. These observations further our understanding of the preclinical disease processes and interplay between microvascular and macrovascular disease.

## Abstract 2

Supplemental Digital Content is available in the text.

Heart disease remains a leading cause of death internationally,^1^ despite advances in the diagnosis and treatment of hypertension and cardiovascular disease (CVD). It has long been thought that the microvasculature may play a pivotal role in the pathogenesis of CVD.^2^ Optimal vascular architecture achieves the most efficient blood flow with minimum energy allowing for maximum vascular diffusion.^3^ Therefore, alterations in geometry of the retinal vasculature may reflect a state of dysfunction of the microvasculature more generally and provide a noninvasive marker of lifetime CVD risk factor load^2,4^ and potentially predict disease development.^2,5^

Retinal arteriolar narrowing has consistently been associated with higher blood pressure (BP)^6–8^ the onset of hypertension^5,9^ and with an increased risk of incident CVD events and mortality.^2^ Evidence is emerging to suggest an association between tortuosity and an increased risk of stroke,^4,8,10^ and a growing body of evidence has suggested associations with arterial stiffness, indicating systemic effects.^11^ To date, limited research has been undertaken to assess the association of retinal vessel tortuosity with BP, and the direction of associations observed with vessel tortuosity have been inconsistent.^4,6,12,13^ Previous smaller studies have shown an increase in vessel tortuosity (both arteriolar and venular tortuosity) with higher BP,^8^ while others have shown an association in the opposite direction or no association at all.^4,6,12–14^

With recent advances in technology, a study by Poplin et al^15^ using a deep learning approach has provided some encouraging evidence on the potential value of risk prediction utilizing retinal imaging at scale. While retinal vessels seem to be key areas of interest, understanding how these models predict risk still remains a substantial problem of the deep learning approach. Retinal vessel morphometry assessment provides insight into the nature of vessel changes, furthering our understanding of potential mechanistic pathways. We examined the association of retinal vessel morphometry (using a fully automated retinal image analysis system)^16^ with blood pressure (BP) and arterial stiffness in United Kingdom Biobank (UKBB).

## Methods

Anonymized data will be made available by the UKBB on request https://www.ukbiobank.ac.uk/scientists-3/. UKBB recruited >500 000 people aged 40 to 69 years, between 2006 and 2010, from across the United Kingdom. A subset of 68 550 participants from 6 UKBB centers had retinal images captured. The Cohort profile: design and methods in the eye and vision consortium of UKBB has recently been published.^17^ The eye and vision substudy has a very similar profile to the main UKBB study. Nonmydriatic fundus cameras (Topcon 3D OCT-1000 Mk 2) with a 45° field-of-view were used to capture color images. Images were saved in PNG format with a resolution of 2048×1536 pixels.

### Physical Examination

Age, sex, and other sociodemographic characteristics (at a postcode level, neighborhood deprivation was expressed in terms of the the Townsend deprivation index, a higher Townsend index score implies a greater degree of deprivation) were collected by questionnaire. Height was measured to the nearest centimeter (cm) using a Seca 202 height measure, and a Tanita BC-418 body composition analyzer was used to measure weight to the nearest 0.1 kg. Body mass index (BMI) was calculated as weight (kilograms) divided by height squared (square meters). Two BP and heart rate measurements were taken with an Omron 705 IT electronic BP monitor, while seated at least 1-minute apart^18^ and the average of the measures used in the analyses. A question on medication for BP was used to define antihypertensive medication usage. Hypertension was defined as having systolic BP≥140 mm Hg, or diastolic BP≥90 mm Hg, or self-reported use of antihypertensive medication. Mean arterial pressure (MAP) was calculated as [(2×diastolic BP)+systolic BP]/3. Mean pulse pressure (PP) was calculated as systolic BP minus diastolic BP. History of myocardial infarction, stroke, and diabetes mellitus were determined by self-report. The HbA1c assay was performed using 5 Bio-Rad Variant II Turbo analyzers. Analysis of serum biomarkers utilized 10 immunoassay analyzers (6× DiaSorin Liaison XL and 4× Beckman Coulter DXI 800 and 4 clinical chemistry analyzers (2× Beckman Coulter AU5800 and 2× Siemens Advia 1800).

### Retinal Imaging and Processing

Retinal images were processed using an automated computerized system (QUARTZ [Quantitative Analysis of Retinal Vessel Topology and Size]).^16^ In brief, the automated system obtained thousands of measures of diameter and tortuosity from the whole retinal image.^16,19,20^ These measures were summarized using mean diameter and tortuosity, weighted by vessel segment length, for arterioles and venules separately for each image. The following image processing modules were all validated on a subset of 4692 retinal images from a random sample of 2346 UKBB participants: including vessel segmentation, image quality score, optic disc detection, vessel width measurement, tortuosity measurement, arteriolar venular recognition.^16,20,21^ The performance of the Arteriole/Venule recognition had detection rates of up to 96% for arterioles and 98% for venules when the automated probability of arteriole or venule was set to a cutoff of 0.8. An automated assessment of image quality was also made based on the segmented vasculature.^21^ The algorithm achieved a sensitivity of 95.3% and a specificity of 91.1% for the detection of inadequate images.^20^ A model eye was used to quantify the magnification characteristics of the Topcon 3D OCT-1000 Mk 2 fundus camera, allowing pixel dimensions of vessel diameter to be converted to real size.^22^

### Arterial Stiffness

Arterial stiffness was measured noninvasively using the pulse waveform obtained at the finger with an infra-red sensor (Pulse Trace PCA2, CareFusion).^23^ Participant’s height (meters) was divided by the time between the peaks of the pulse waveform to obtain the Arterial Stiffness Index (ASI) in meters per second.

The UKBB study was approved by the Northwest Region NHS research ethics committee.

### Statistical Analysis

Stata 15.0 IC (Stata Corp LP, College Station, TX) was used to analyze the data. Retinal vessel diameters were normal distributed, measures of tortuosity were positively skewed, and therefore log-transformed. Multilevel linear regression models adjusting for age, sex, ethnicity, and UKBB center as fixed effects, with a random effect for person to allow for repeated measures of vessel indices within the same person (model 1), were used to examine associations of cardiometabolic risk factors with retinal vessel outcomes. Model 2 extended model 1 with further adjustment for smoking, Townsend deprivation index, and BMI. Model 3 included the same factors as model 2 with additional adjustment for total cholesterol, triglycerides, and HbA1c and exclusion participants with a history of myocardial infarction, stroke, diabetes mellitus, on treatment for hypertension or who answered unknown or declined to answer. Data missing on categorical variables were included as an additional category for each variable, to minimize data loss. Associations with the log-transformed tortuosity were exponentiated to give percentage differences in tortuosity and absolute differences (in microns) in vessel width per specified increase with CVD risk markers. Associations between retinal markers and each CVD risk factor were examined for interactions with sex; interactions were considered statistically significant if the *P* for interaction were <0.01 given the large sample size of UKBB.

## Results

**Figure 1.**
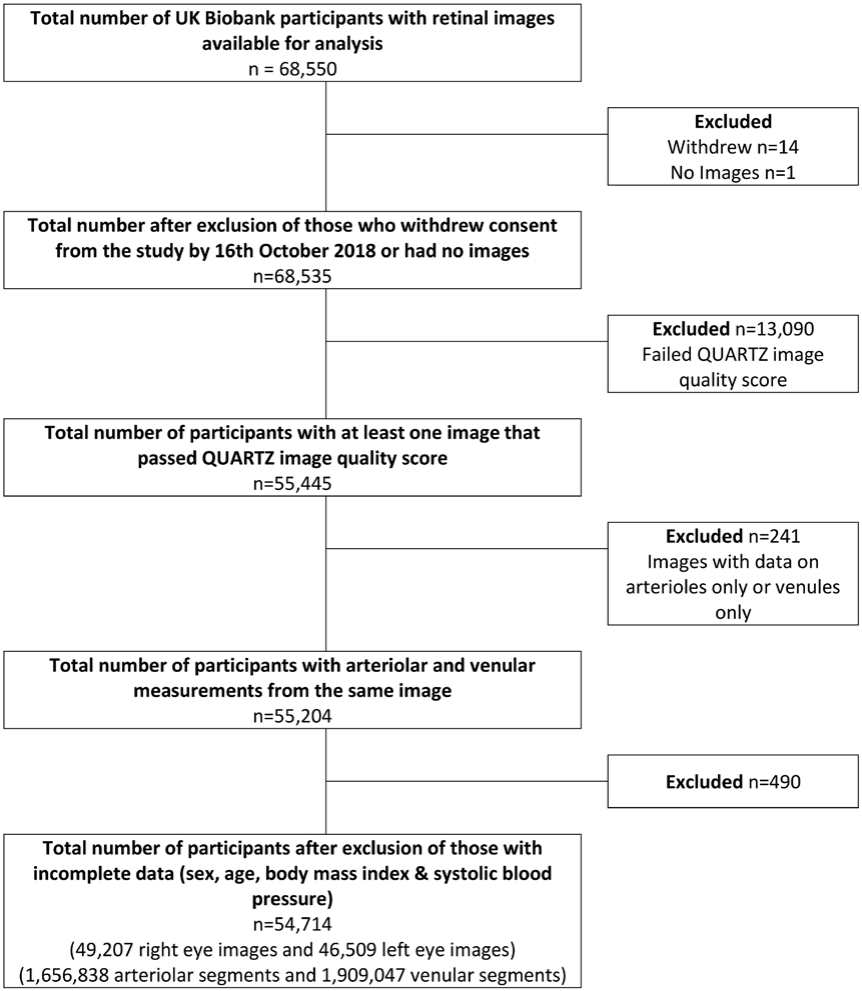
Flow of participants by exclusion from the United Kingdom Biobank retinal imaging study. QUARTZ indicates Quantitative Analysis of Retinal Vessel Topology and Size.

**Figure 2.**
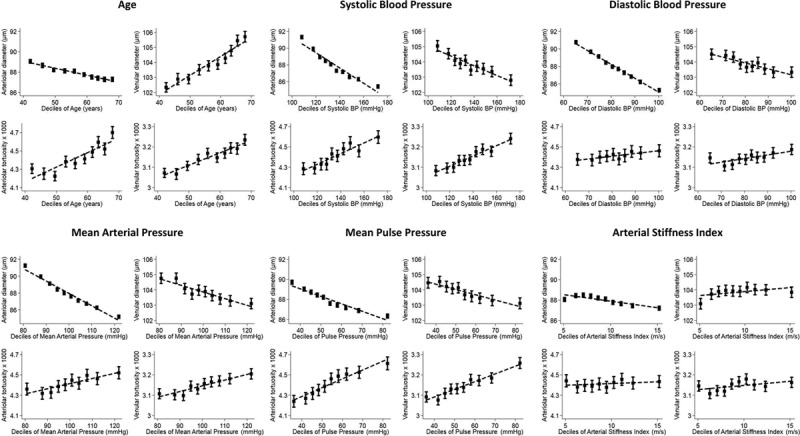
Adjusted mean retinal vessel width and tortuosity by deciles of cardiovascular disease (CVD) risk factors. Adjusted means (solid square symbols), 95% CIs (solid vertical error bars) and regression line (dotted line) are from a multilevel model allowing for age, sex, and CVD risk maker decile as fixed effects and repeated retinal vessel measures within each person. BP indicates blood pressure.

A total of 54 714 participants (79.8% of UKBB participants who underwent fundus imaging) were included in these analyses (Figure 1). Population characteristics are shown in Table 1. Figure 2 shows adjusted mean retinal vessel measures (adjusted for age, sex, and random effect for person), by risk factor deciles.

**Table 1.**
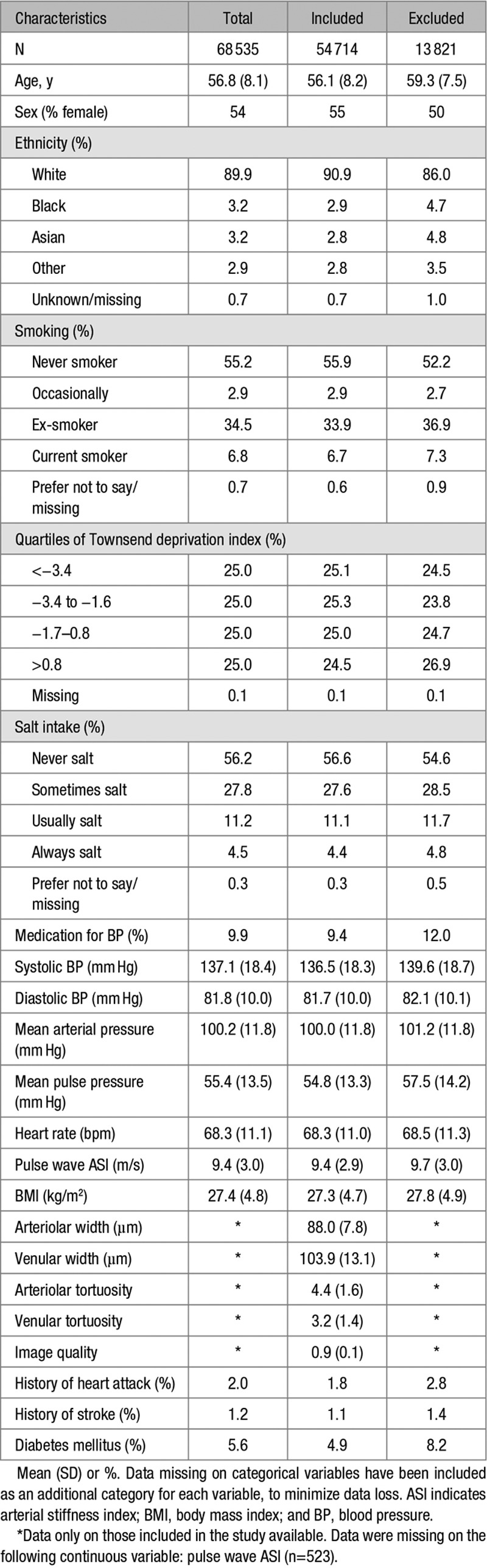
Characteristics of the Population by Inclusion and Exclusion Criteria

### Retinal Tortuosity, BP, and ASI Measures

Increased arteriolar tortuosity was associated with higher systolic BP, diastolic BP, MAP and PP, after adjustment (Table 2, model 1). For example, a 10 mmHg higher systolic BP was associated with a 1.2% (95% CI, 0.9; 1.4%; *P*=6.4×10^−20^) rise in arteriolar tortuosity. Associations observed were unaffected by additional adjustments in model 2 or after removal of participants with history of CVD and with adjustment for total cholesterol, triglycerides, and HbA1c (model 3—Table S1 in the online-only Data Supplement). Increased retinal venular tortuosity was associated with higher systolic BP, diastolic BP, MAP, PP, and ASI (model 1). For example, a 10 mmHg rise in PP was associated with a 1.1% (0.8%–1.3%) rise in venular tortuosity. Adjustment for factors in model 2 marginally attenuated the association of venular tortuosity with systolic BP and PP, but each association remained highly statistically significant. However, associations were no longer significant for MAP or ASI. Removal of participants with history of CVD (model 3, Table S1) and adjustment for total cholesterol, triglycerides, and hbA1c did not alter these associations but the association with diastolic BP was no longer statistically significant. The association of increased tortuosity (both arteriolar and venular tortuosity) with higher systolic BP remained after adjustment for retinal diameters.

**Table 2.**
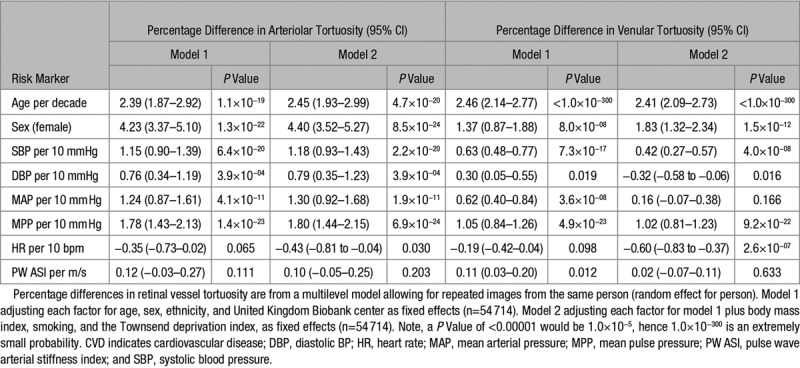
Percentage Differences in Arteriolar and Venular Tortuosity Associated With CVD Risk Factors

### Retinal Diameters BP and ASI Measures

Narrower arterioles were associated with higher systolic BP, diastolic BP, MAP, PP, heart rate, and ASI (Table 3, model 1). For example, a unit increase in ASI was associated with a −0.11 µm (−0.13, −0.09 µm; *P*=8.59×10^−23^) decrease in arteriolar diameter, which would equate approximately to 0.33 µm narrowing per SD increase in ASI. Adjustments in model 2 had no impact on the association of narrower arterioles with higher systolic BP, diastolic BP, MAP, PP, heart rate, and ASI. Additional adjustment for systolic BP had little impact on the association of ASI with arteriolar diameter. The associations with CVD risk factors were unaffected by removal of those with history of CVD or adjustment for total cholesterol, triglycerides, and HbA1c (model 3, Table S2). Narrower venules were also associated with higher systolic BP, diastolic BP, MAP, and PP. Wider venules were associated with increased ASI and heart rate (model 1, Table 3). Adjustment for additional confounding (model 2) did not alter the associations (with diameters), except for ASI which was no longer significantly associated with wider venules. Associations were unaffected by removal of event history or adjustment for total cholesterol, triglycerides, and HbA1c (model 3; Table S2).

**Table 3.**
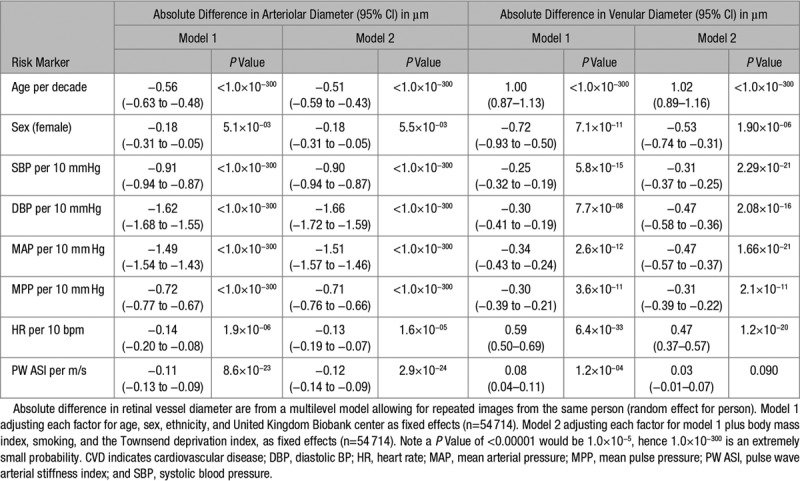
Mean Difference in Arteriolar and Venular Diameter (micrometers) Associated With CVD Risk Factors

Although females exhibited narrower and more tortuous vessels, formal tests of interaction of all associations with sex were not statistically significant. Out of 36 tests for interaction performed, 6 were statistically significant at the 1% level. However, the absolute differences in slopes were minimal, and all of the associations were in the same direction in men and women (data available on request). None of the associations were in opposing directions and the results are presented for men and women combined.

## Discussion

In this study, the first to examine the retinal microvasculature at scale using fully automated software (with over 3.5 million vessel segments from over 50 000 participants), we have shown novel associations between retinal microvascular tortuosity with BP and ASI and confirmatory associations with diameters. Each significant risk factor expressed in deciles (Figure 2) showed strong graded associations with retinal vessel morphometry measures. The associations held after adjustment for confounding factors and removal of those with self-reported diabetes mellitus and CVD morbidity. Importantly these morphometric associations may be indicative of preclinical disease processes, suggesting a role in CVD risk prediction.

### Retinal Tortuosity and CVD Risk Factors

The key findings from the present study were that increased retinal tortuosity (arteriolar and venular) showed strong graded associations with higher BP, MAP, and PP, with *P* as small as 1×10^−300^. The retinal microvasculature abnormalities observed with increased BP have not been so clear until now (Figure 2). The European Prospective Investigation into Cancer-Norfolk Eye study of 5947 participants (which used an identical methodology) showed remarkably similar increased tortuosity with systolic BP in older adults (arteriolar tortuosity, 1.2%; 95% CI, 0.5; 1.9% per 10 mmHg and venular tortuosity, 0.5%; 95% CI, 0.02; 0.88% per 10 mmHg).^8^ However, evidence from other smaller scale studies have been less supportive. A nested case-control study by Witt et al^4^ among 682 adults showed an increase in arteriolar tortuosity was weakly associated with higher systolic BP, with no evidence of an association with venular tortuosity. In contrast, a cross-sectional study by Cheung et al^14^ showed a decrease in arteriolar tortuosity with higher BP and MAP and an increase in venular tortuosity with higher BP and MAP, in an Asian population of adults (n=2915). Disagreements between studies may be because of smaller sample sizes together with measurement error (particularly with methods relying on manual measurement), where there is less certainty over the presence or absence of underlying associations.

While tortuosity (arteriolar and venular) was higher overall among women compared with men, in the present study, the association between CVD risk markers and tortuosity was evident in both sexes (and independent of further adjustment for height). Overall, the association of increased tortuosity (both arteriolar and venular tortuosity) with higher BP in the present study was independent of retinal diameters. This suggests that tortuosity reflects different structural vascular changes from those shown for diameters and may provide additional value to CVD risk prediction tools beyond the current testing of diameters.^24^

The association of retinal tortuosity with stroke has not been well studied, although 2 studies have shown a more tortuous retinal microvascular network with prevalent stroke.^8,10^ In UKBB, there was no evidence of an association between self-reported stroke and arteriolar tortuosity. This difference may be related to differences in age (UKBB participants are relatively younger) and case ascertainment of stroke. In UKBB, presence of stroke was based on self-report, while in the other studies it was based on clinical information from health records.^8,10^ Conversely, in the present study, increased venular tortuosity was strongly associated with stroke for which some evidence has been reported previously.^8,10^ The discrepancies overall in findings from earlier studies with tortuosity may relate to the limited sample sizes, characteristics of the population (ie, age and risk factor profile, differences in diabetes mellitus duration, and type) or the time placement of the study. For example, treatment has improved over time and therefore it may be harder to observe changes associated with a history of events in more recent studies compared with studies from 10 to 20 years ago. Given the strong graded association of CVD risk factors with tortuosity (both arteriolar and venular) in the present study of over 50 000 participants and the replication of the findings observed in EPIC,^8^ the associations between tortuosity and CVD risk factors are now very clear warranting verification in a large longitudinal follow-up study, to confirm causality.

### Retinal Diameters and CVD Risk Factors

Retinal arteriolar narrowing has consistently been associated with elevated BP in epidemiological studies^6–8,25^ and meta-analysis,^9^ supporting the finding of the present study (Figure 2), where each 10 mmHg increase in systolic BP was associated with a 0.9 µm narrowing in arteriolar diameter. The weaker association observed for venular diameter in the present study and no association observed in smaller previous studies^6,7^ suggests that the impact of BP on arterioles is more prominent than on venules. In general, the literature is in agreement that arterioles and venules are differentially associated with cardiometabolic risk factors; narrower arterioles are associated with higher BP^8^, and wider venules with inflammation and higher BMI/obesity.^8,26^ This most likely relates to the effect of different mechanistic pathways, which are yet to be fully understood.

We identified a strong association between older age and venular widening (Figure 2) and arteriolar narrowing. The aging process has been linked to decreased retinal vessel density, reduced inner retinal layer thickness, and retinal blood flow velocity, particularly in venules.^27^ The sex difference, with females having narrower arteriolar and venular diameters compared with males would need to be considered in the development of CVD risk prediction tools.

### Future Direction—Fully Automated CVD Risk Prediction

The work by Poplin et al^15^ using a convolutional neural network was able to predict CVD risk factors and outcomes from a retinal image alone as accurately as available CVD risk prediction tools. In support of growing evidence, the present study has shown that narrower arteriolar diameters are associated with increased BP, ASI, and PP very strongly and provide an indication of systemic microvascular and macrovascular changes.^11^ Understanding how models predict risk remains a substantial problem of the deep learning, which is very much a black box approach; therefore, assessment of vessel morphology coupled with convolutional neural network deep learning approaches may help further inform potential pathways of disease processes and prediction.

The strength of this study includes its large sample size of over 50 000 participants. The QUARTZ software is fully automated, incorporates convolutional neural network technology, and utilizes information from all vessels extracted within an image, providing precise measurement. A potential limitation is the use of the entire retinal image compared with a section of the image used by other grading systems; however, given our findings are consistent with previous literature, this is unlikely to be a major issue. The study limitations include the cross-sectional study design, meaning the issue of causality cannot be resolved until further follow-up. Although the UKBB is not a representative sample of the United Kingdom population, which is an issue if one were attempting to determine prevalence of outcomes or genetic associations in particular.^28^ However, this study was focused on assessing cross-sectional phenotypic associations, and Pizzi et al^29^ showed that using restricted sampling for a cohort study is unlikely to appreciably bias estimates of exposure-disease associations, under a range of potential scenarios. In support of this, our findings show very similar patterns of association to those observed in an entirely independent United Kingdom-based cohort study of similar and older age (the EPIC-Norfolk study).^8^ The impact of hypertension treatment duration could not be assessed, so we are unsure of the impact of treatment duration on the association between retinal measures and BP, though we did note that removing those on BP treatment did not alter the associations observed. The PWV device used a nonreference method to estimate PWV, which is not as accurate as other approaches and may have led to an underestimation of the association, particularly given that pulse pressure shows stronger associations than PWV in this study. However, reassuringly, the association is in the expected direction and a *P* of 2.9×10^−24^ is highly unlikely to be a chance finding. This lends weight to the null association observed between PWV and vessel tortuosity

### Perspectives

This study assessing the retinal microvasculature at scale has shown clear associations between retinal vessel morphometry, BP, and ASI. These observations further our understanding of the preclinical disease processes and interplay between microvascular and macrovascular disease.

## Sources of Funding

The project was funded by a grant from the British Heart Foundation (PG/15/101/31889). This research has been conducted using the United Kingdom Biobank Resource under Application Number 522. The list of UK Biobank Eye and Vision Consortium members is available from the consortium website (http://www.ukbiobankeyeconsortium.org.uk/people).

## Disclosures

None.

## Supplementary Material

## Novelty and Significance

### What Is New

- This is the first study to examine the retinal microvasculature at scale using fully automated software (with over 3.5 million vessel segments from over 50 000 participants), taking retinal morphology assessment from just a research tool to having potential use within clinical practice.
- The observations from this study further our understanding of the preclinical disease processes, and relationship between microvascular and macrovascular changes.

### What Is Relevant?

- There are clear linear associations between retinal vessel morphometry measures and blood pressure and arterial stiffness index.
- Associations held after adjustment for confounding factors and removal of those with self-reported diabetes mellitus and cardiovascular disease morbidity.

### Summary

- This study assessing the retinal microvasculature at scale has shown clear associations between retinal vessel morphometry, blood pressure, and arterial stiffness index.
- Importantly, these morphometric associations may be indicative of preclinical disease processes, suggesting a role in cardiovascular disease risk prediction.
