# Supplementary material for: Associations of Retinal Microvascular Diameters and Tortuosity With Blood Pressure and Arterial Stiffness: United Kingdom Biobank
Source: Hypertension. 2019 Oct 28;74(6):1383–90. doi: 10.1161/HYPERTENSIONAHA.119.13752 (PMC7069386; doi:10.1161/HYPERTENSIONAHA.119.13752)
Supplement: Supplementary file 1 [file hyp-74-1383-s001.docx]

**Online Supplement**

**Associations of Retinal Microvascular Diameters and Tortuosity with Blood Pressure and Arterial Stiffness: UK Biobank**

**Running Title: Retinal Microvascular Morphology and Blood Pressure**

Robyn J Tapp, PhD^1^

Christopher G Owen, PhD^1^

Sarah A Barman, PhD^2^

Roshan A Welikala, PhD^2^

Paul J Foster, PhD^3 & 4^

Peter H Whincup, FRCP^1^

David P Strachan, MD^1^

Alicja R Rudnicka, PhD^1^

For the UK Biobank Eye & Vision Consortium

^1^Population Health Research Institute, St George’s, University of London, United Kingdom

^2^Faculty of Science, Engineering and Computing, Kingston University, Surrey, United Kingdom

^3^Integrative Epidemiology Research Group, UCL Institute of Ophthalmology, United Kingdom

^4^NIHR Biomedical Research Centre at Moorfields Eye Hospital, United Kingdom

**Correspondence to:**

Professor Alicja Rudnicka

Population Health Research Institute

St George’s, University of London, United Kingdom

Cranmer Terrace

London SW17 0RE

Tel 0208 725 2799

Email: [arudnick@sgul.ac.uk](mailto:arudnick@sgul.ac.uk)

| **Table S1. Percentage differences in arteriolar and venular tortuosity associated with CVD risk factors** | | | | | |
| --- | --- | --- | --- | --- | --- |
|  | **Percentage Difference in Arteriolar Tortuosity (95%CI)** | |  | **Percentage Difference in Venular Tortuosity (95%CI)** | |
|  | **Model 3** | |  | **Model 3** | |
| **Risk marker** |  | **P value** |  |  | **P value** |
|  |  |  |  |  |  |
| Age per decade | 2.21 (1.58, 2.84) | 3.1E-12 |  | 2.41 (2.04, 2.78) | 3.6E-38 |
| Sex (female) | 4.29 (3.25, 5.34) | 2.0E-16 |  | 1.52 (0.92, 2.12) | 5.5E-07 |
| SBP per 10mmHg | 1.31 (1.01, 1.62) | 3.2E-17 |  | 0.46 (0.28, 0.64) | 5.3E-07 |
| DBP per 10mmHg | 0.92 (0.38, 1.46) | 8.0E-04 |  | -0.33 (-0.64, -0.01) | 0.042 |
| MAP per 10 mmHg | 1.46 (1.00, 1.93) | 6.0E-10 |  | 0.19 (-0.08, 0.46) | 0.171 |
| MPP per 10 mmHg | 2.00 (1.57, 2.43) | 5.6E-20 |  | 1.11 (0.86, 1.36) | 5.3E-18 |
| HR per 10 bpm | -0.58 (-1.06, -0.11) | 0.017 |  | -0.80 (-1.08, -0.52) | 2.4E-08 |
| PW ASI | 0.08 (-0.10, 0.26) | 0.392 |  | 0.02 (-0.08, 0.13) | 0.688 |
| Percentage differences in retinal vessel tortuosity are from a multilevel model allowing for repeated images from the same person (random effect for person). Confidence interval (CI). Model 3 adjusting for each factor for model 2 plus HbA1c, total cholesterol and triglycerides and excluding those with diabetes, heart attack, stroke, on medication for BP or who answered unknown or declined to answer (n=38,487). NB A p value of <0.00001 would be 1.0E-5 hence 1.0E-300 is an extremely small probability. SBP – systolic blood pressure, DBP diastolic BP, PW ASI – pulse wave arterial stiffness index, HR – heart rate, MAP mean arterial pressure, MPP – mean pulse pressure. | | | | | |
|  |  |  |  |  |  |
|  |  |  |  |  |  |
|  |  |  |  |  |  |

| **Table S2. Mean difference in arteriolar and venular diameter (µm) associated with CVD risk factors** | | | | | |
| --- | --- | --- | --- | --- | --- |
|  | **Absolute Difference in Arteriolar Diameter (95%CI) in µm** | |  | **Absolute Difference in Venular Diameter (95%CI) in µm** | |
|  | **Model 3** | |  | **Model 3** | |
| **Risk marker** |  | **P value** |  |  | **P value** |
|  |  |  |  |  |  |
| Age per decade | -0.56 (-0.65, -0.47) | 5.0E-33 |  | 0.96 (0.80, 1.12) | 2.5E-33 |
| Sex (female) | -0.13 (-0.28, 0.02) | 0.082 |  | -0.49 (-0.75, -0.24) | 1.5E-04 |
| SBP per 10mmHg | -0.94 (-0.98, -0.90) | <1.0E-300 |  | -0.31 (-0.39, -0.23) | 3.6E-15 |
| DBP per 10mmHg | -1.74 (-1.81, -1.66) | <1.0E-300 |  | -0.47 (-0.61, -0.34) | 1.0E-11 |
| MAP per 10 mmHg | -1.58 (-1.65, -1.51) | <1.0E-300 |  | -0.47 (-0.59, -0.35) | 3.2E-15 |
| MPP per 10 mmHg | -0.75 (-0.81, -0.68) | <1.0E-300 |  | -0.31 (-0.42, -0.20) | 2.0E-08 |
| HR per 10 bpm | -0.13 (-0.21, -0.06) | 2.7E-04 |  | 0.49 (0.37, 0.61) | 4.6E-15 |
| PW ASI | -0.11 (-0.14, -0.08) | 7.6E-16 |  | 0.03 (-0.02, 0.07) | 0.268 |
| Absolute difference in retinal vessel diameter are from a multilevel model allowing for repeated images from the same person (random effect for person). Confidence interval (CI). Model 3 adjusting for each factor for model 2 plus HbA1c, total cholesterol and triglycerides and excluding those with diabetes, heart attack, stroke, on medication for BP or who answered unknown or declined to answer (n=38,487). NB A p value of <0.00001 would be 1.0E-5 hence 1.0E-300 is an extremely small probability. SBP – systolic blood pressure, DBP - diastolic BP, PW ASI – pulse wave arterial stiffness index, HR – heart rate, MAP - mean arterial pressure, MPP – mean pulse pressure. | | | | | |
|  |  |  |  |  |  |
|  |  |  |  |  |  |
