# Supplementary figures and images for: Associations of Retinal Microvascular Diameters and Tortuosity With Blood Pressure and Arterial Stiffness: United Kingdom Biobank
Source: Hypertension. 2019 Oct 28;74(6):1383–90. doi: 10.1161/HYPERTENSIONAHA.119.13752 (PMC7069386; doi:10.1161/HYPERTENSIONAHA.119.13752)

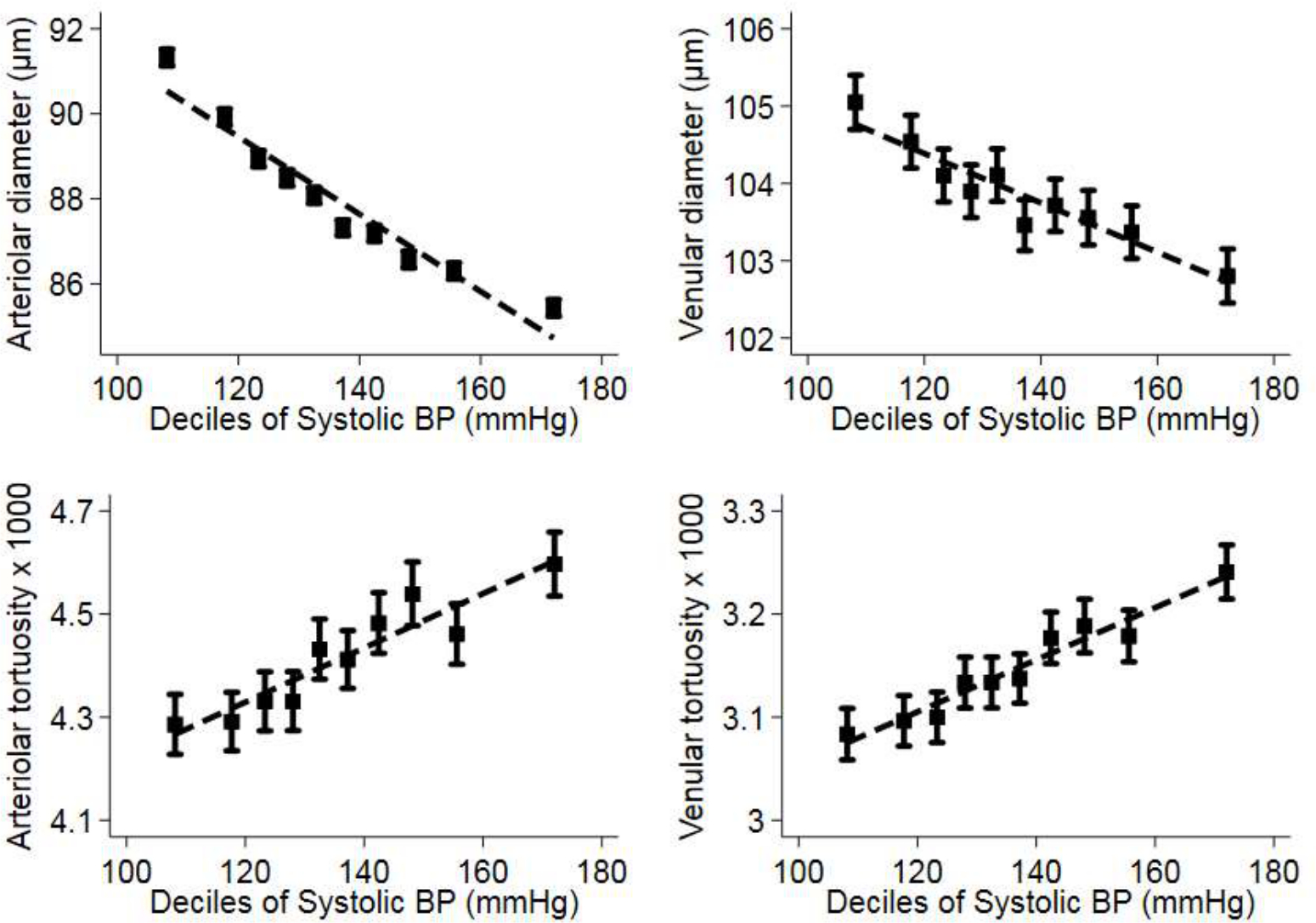

Supplement: Supplementary file 2 [file hyp-74-1383-s002.jpg]
